# Expression of a Bacterial Trehalose-6-phosphate Synthase otsA Increases Oil Accumulation in Plant Seeds and Vegetative Tissues

**DOI:** 10.3389/fpls.2021.656962

**Published:** 2021-03-10

**Authors:** Zhiyang Zhai, Jantana Keereetaweep, Hui Liu, Regina Feil, John E. Lunn, John Shanklin

**Affiliations:** ^1^Department of Biology, Brookhaven National Laboratory, Upton, NY, United States; ^2^Max Planck Institute of Molecular Plant Physiology, Potsdam-Golm, Germany

**Keywords:** trehalose 6 phosphate, triacylglycerol, fatty acid synthesis, oil accumulation, sugar signaling

## Abstract

We previously demonstrated that exogenous trehalose 6-phosphate (T6P) treatment stabilized WRINKLED1 (WRI1), a master transcriptional regulator of fatty acid (FA) synthesis and increased total FA content in *Brassica napus* (*B. napus*) embryo suspension cell culture. Here, we explore *Arabidopsis* lines heterologously expressing the *Escherichia coli* T6P synthase (otsA) or T6P phosphatase (otsB) to refine our understanding regarding the role of T6P in regulating fatty acid synthesis both in seeds and vegetative tissues. *Arabidopsis* 35S:*otsA* transgenic seeds showed an increase of 13% in fatty acid content compared to those of wild type (WT), while seeds of 35:*otsB* transgenic seeds showed a reduction of 12% in fatty acid content compared to WT. Expression of otsB significantly reduced the level of WRI1 and expression of its target genes in developing seeds. Like *Arabidopsis* seeds constitutively expressing otsA, transient expression of otsA in *Nicotiana benthamiana* leaves resulted in strongly elevated levels of T6P. This was accompanied by an increase of 29% in *de novo* fatty acid synthesis rate, a 2.3-fold increase in triacylglycerol (TAG) and a 20% increase in total fatty acid content relative to empty vector (EV) controls. Taken together, these data support the heterologous expression of otsA as an approach to increasing TAG accumulation in plant seeds and vegetative tissues.

## Introduction

Lipids play key roles as structural components of cell membranes, energy-dense storage compounds, and cell signaling molecules. Fatty acids (FA) are major components of triacylglycerols (TAG), which occur in all tissues but accumulate to very high levels within lipid droplets in plant seeds (Li-Beisson et al., 2013). *De novo* synthesis of FA occurs in the plastid *via* the action of two multi-component enzyme systems: acetyl-CoA carboxylase (ACCase) and fatty acid synthase (Ohlrogge and Browse, 1995; Rawsthorne, 2002). In *Arabidopsis*, WRINKLED1 (WRI1), an APETALA2 (AP2)-type transcription factor, is a master transcriptional activator of FA synthesis. To date, more than 20 *WRI1* target genes coding for various steps in glycolysis and fatty acid synthesis have been identified (Ruuska et al., 2002; Baud et al., 2007; Maeo et al., 2009; Fukuda et al., 2013; Li et al., 2015; Liu et al., 2019).

Fatty acids are synthesized *de novo* from acetyl-CoA, which is ultimately derived from photosynthetically derived sugars. Previous studies have demonstrated that sugars can potentiate fatty acid synthesis. For example, *Arabidopsis* leaves in which *ADG1* (encoding the small subunit of ADP-glucose pyrophosphorylase) was reduced by RNAi contained 3-fold more sucrose along with a 30% increase in TAG relative to wild type (WT; Sanjaya et al., 2011). In another study, it was shown that *Arabidopsis* roots accumulated 4-fold more TAG in the presence of one-half-strength MS medium supplemented with 5% sucrose compared to controls lacking sucrose (Kelly et al., 2013). To test the influence of endogenous sugar content on FA and TAG accumulation, we generated a high-leaf-sugar line by reducing sugar phloem loading along with starch synthesis by crossing the *suc2* (encoding a sucrose/H+ symporter that loads Sucrose into the phloem) mutant (Srivastava et al., 2009) with the *adg1* mutant. The sugar content (combined glucose and sucrose) in *adg1suc2* leaves is 80-fold higher than that of WT. Leaf TAG accumulation in *adg1suc2* increased by more than 10-fold relative to WT reaching approximately 1% of dry weight (DW; Zhai et al., 2017b). Besides the effects of sugars as direct precursors that supply carbon skeletons for FA synthesis, progress has also been made toward elucidating the effects of sugar signaling on FA synthesis. In one example, pyruvate and the tricarboxylic acid cycle intermediates: 2-oxoglutarate and oxaloacetate were shown to completely reverse the PII-dependent inhibition of ACCase (Bourrellier et al., 2010). PII is an evolutionarily conserved signal integrator involved in the regulation of nitrogen/carbon homeostasis in bacteria and plants that binds to the biotin carboxyl carrier protein (BCCP) subunit of the plastidial ACCase inhibiting its activity by up to 50% (Bourrellier et al., 2010). A second link between the availability of sugar and FA synthesis involves the snf1-related protein kinase1 (SnRK1), a major plant carbon/energy sensor (Baena-González et al., 2007). Under low sugar conditions, KIN10, the catalytic subunit of SnRK1 phosphorylates WRI1 predisposing it to proteasomal degradation. However, in the presence of higher sugar levels, KIN10 phosphorylation of WRI1 is inhibited and WRI1 is stabilized, increasing the transcription of WRI1 target genes involved in FA synthesis (Zhai et al., 2017a). This regulatory mechanism, couples FA synthesis to the availability of cellular carbon and energy.

In plants, the phosphorylated disaccharide trehalose 6-phosphate (T6P) acts as a signal of sucrose availability connecting its intracellular metabolic status with plant growth and development (Schluepmann et al., 2003; Lunn et al., 2006; Yadav et al., 2014; Figueroa and Lunn, 2016; Fichtner and Lunn, 2021). T6P is synthesized by the action of T6P synthase (TPS) with UDP-Glc (UDPG) and Glc 6-phosphate (G6P), both of which are central to plant metabolism (Cabib and Leloir, 1958). SnRK1 activity in crude extracts from developing *Arabidopsis* tissues is strongly inhibited by T6P, and the inhibition was reported to depend on unknown protein factor(s) principally expressed in young tissues (Zhang et al., 2009; Martínez-Barajas et al., 2011; Griffiths et al., 2016). KIN10 is activated by GEMINIVIRUS REP-INTERACTING KINASE1 (GRIK1; also known as SnRK1 ACTIVATING KINASE1, SnAK1) and GRIK2 (SnAK2), which phosphorylate T175 in KIN10’s activation loop (Shen et al., 2009; Glab et al., 2017).

We recently demonstrated that T6P can bind directly to KIN10 at physiological concentrations and weaken its association with GRIK, thereby lowering its activation status and inhibiting SnRK1 phosphorylation of WRI1. This was confirmed by *in vivo* experiments in which a *Brassica napus* suspension cell culture was fed T6P in the medium, and WRI1 accumulated, resulting in the activation of FA synthesis (Zhai et al., 2018).

An alternative to feeding cell cultures with T6P is the use of *Arabidopsis* lines heterologously expressing the *Escherichia coli* T6P synthase (otsA), which have well documented elevation in T6P levels, and lines expressing T6P phosphatase (otsB) to increase T6P dephosphorylation to trehalose, which has been reported to lower T6P levels (Schluepmann et al., 2003; Wingler et al., 2012) or lead to the accumulation of sucrose and a reduction in the T6P:sucrose ratio (Yadav et al., 2014). Here, we make use of these previously reported stably transformed *Arabidopsis* otsA/otsB-overexpressing lines (Schluepmann et al., 2003; Wingler et al., 2012) along with transient expression of otsA and otsB in *Nicotiana benthamiana* leaves to investigate the effects of T6P on the regulation of FA synthesis in seeds and vegetative tissues, respectively. *Arabidopsis* otsA transgenics accumulate more seed oil and tobacco leaves transiently expressing otsA also show increased TAG accumulation, demonstrating that otsA overexpression is a viable approach for increasing lipid accumulation in both source and sink tissues.

## Materials and Methods

### Plant Materials and Growth Conditions

*Arabidopsis 35S:otsA* and *35S:otsB* lines were obtained from Astrid Wingler (University of Cork, Ireland; Schluepmann et al., 2003; Wingler et al., 2012). *Arabidopsis* seeds were surface-sterilized and selected on agar plates containing half-strength Murashige and Skoog salts. After 1 week, seedlings were transplanted to moist soil (seed BM2 mix, Berger, Saint-Modeste, Canada). All plants (*Arabidopsis* and *N. benthamiana*) were grown with a 16 h-light/8 h-dark photoperiod (combination of cool white, fluorescent lamps, and incandescent lamps, at a photosynthetic photon flux density of 250 μmol m^−2^ s^−1^) with a 23°/19°C day/night, 16/8 h temperature regime and approximately 75% relative humidity.

### Genetic Constructs

The *otsA* and *otsB* coding regions were amplified by PCR from *E. coli* genomic DNA using primer pairs listed in supplementary Table S1. The PCR products were then cloned into the Invitrogen GATEWAY^M^ pDONR/Zeo vector (Thermo Fisher Scientific, Waltham, MA)^1^ using the BP reaction and sub-cloned (LR reaction) into the plant GATEWAY™ binary vector: pGWB414 (Nakagawa et al., 2007) for transient expression in *N. benthamiana*.

### Agroinfiltration of *Nicotiana benthamiana*

Transient gene expression in *N. benthamiana* by agroinfiltration was accomplished using a previously described procedure (Ohad and Yalovsky, 2010). Infiltrated leaves were harvested 3 days after infiltration with different constructs and analyzed for T6P and lipid contents and for SnRK1 kinase activity and *in vivo* [1-^14^C] acetate labeling.

### T6P Quantification

Water-soluble metabolites were extracted from aliquots (10–20 mg) of frozen tissue powder using chloroform-methanol (Lunn et al., 2006) and evaporated to dryness using a centrifugal vacuum drier. The dried extract was dissolved in 350 μl purified H_2_O and filtered through MultiScreen PCR-96 Filter Plate membranes (Merck Millipore)^2^ to remove high molecular weight compounds. T6P, phosphorylated intermediates, and organic acids were measured by high performance anion-exchange chromatography coupled to tandem mass spectrometry as described by Lunn et al. (2006), with modifications as described by Figueroa et al. (2016).

### Triacylglycerol and Total Fatty Acid Quantification

Total lipids (TAG plus polar lipids) were isolated from 100 mg of freshly harvested leaf tissue by the addition of 700 μl of methanol:chloroform:formic acid (2:1:0.1, by volume) by vigorous shaking for 30 min, after which 1 ml of 1 M KCl, 0.2 M H_3_PO_4_ was added. After mixing, the samples were centrifuged at 1,500 × *g* (4°C) for 5 min, and total lipids were collected in the lower phase (chloroform). For TAG quantification, 60 μl of total lipid were separated by Silica Gel 60 (Merck Millipore, Billerica, MA)^3^ TLC developed with hexane:diethyl ether:acetic acid (70,30:1, by volume) and visualized by spraying with 0.05% (w/v) primuline [in 80% (v/v) acetone]. TAG fractions identified under UV light were scraped from the plate and transmethylated to FA methyl esters (FAMEs) by incubation in 1 ml 12% (w/v) boron trichloride in methanol at 85°C for 40 min. For total FA quantification, 10 μl of total lipids were directly transmethylated with boron trichloride-methanol as described above. For both assays, 5 μg heptadecanoic acid (C17:0) was added as internal standard prior to transmethylation. FAMEs were extracted into hexane and dried under a nitrogen stream before being dissolved in 100 μl hexane and analyzed by GC-MS with an Agilent Technologies (Santa Clara, CA)^4^ 7890A GC System equipped with an Agilent 60 m DB23 capillary column (ID 250-μm) and a 5975C mass selective detector.

### *In vivo* [1-^14^C] Acetate Labeling

Labeling experiments were performed essentially as described by Koo et al. (2004). *Nicotiana benthamiana* leaves were incubated in 25 mM MES-NaOH, pH 5.7 buffer containing 0.01% (w/v) Tween-20 as wetting agent under illumination (180 μmol m^−2^ s^−1^) at 25°C. Labeling was initiated by the addition of 370 kBq of sodium [1-^14^C] acetate solution (2.15 GBq/mmol, American Radiolabeled Chemicals, St Louis, MO).^5^ Labeling was terminated by removal of the medium from the leaf, and the sample was washed three times with water. Total lipids were extracted and separated as described above. Radioactivity associated with total lipids was determined by liquid scintillation counting using a Tri-carb instrument (PerkinElmer).

### Antibodies and Immunoblotting

Anti-WRI1 polyclonal antibodies were described by Zhai et al. (2017a). Anti-histone H3 polyclonal antibodies were purchased from Agrisera (Catalog No. AS10710, Vännäs, Sweden).^6^ Proteins were resolved by SDS-PAGE (5–15% acrylamide gels) and transferred to PVDF membrane for immunoblot analysis. During primary antibody probe, WRI1 antibody with 1:5,000 dilution or H3 antibody (1:2,000) was incubated at 4°C overnight. Immunoblots of targeted proteins were visualized using alkaline phosphatase-conjugated secondary antibodies with colorimetric detection using 5-bromo-4-chloro-3-indolylphosphate/nitro-blue tetrazolium (BCIP/NBT; Bio-Rad). Immunoblot signals were digitalized with Image Quant LAS4000 and quantified with GelAnalyzer2010a.

### RNA Isolation and Quantitative PCR (RT-qPCR)

To quantify gene expression, total RNA was extracted using an RNeasy Plant Mini Kit (Qiaqen, Gaithersburg, MD)^7^ following the manufacturer’s instructions. cDNA was prepared using SuperScript III First-Strand Synthesis SuperMix (Invitrogen). Quantitative PCR (qPCR) was performed using the CFX96 qPCR Detection System (Bio-Rad) and gene-specific primers for *BCCP2* (At5g15530), *KAS1* (At5g46290), and *PKPβ1* (At5g52920), with *F-box* (At5g15710) for *Arabidopsis*; Ctg24993647 for *N. benthamiana* as a reference gene, using oligonucleotide primers as described in Supplementary Table S1. Statistical analysis of RT-qPCR data was performed using the REST2009 algorithm (Pfaffl et al., 2002).

### Accession Numbers

Sequence data from this article can be found in The *Arabidopsis* Information Resource or UniProtKB under the following accession numbers: *WRI1* (At3g54320), *F-box* (At5g15710), *BCCP2* (At5g15530), *KAS1* (At5g46290), *PKPβ1* (At5g52920), *otsA* (P31677), and *otsB* (C1KFX6).

## Results

### Overexpression of otsA Increases Fatty Acid Content in *Arabidopsis* Seeds

**Figure 1:**
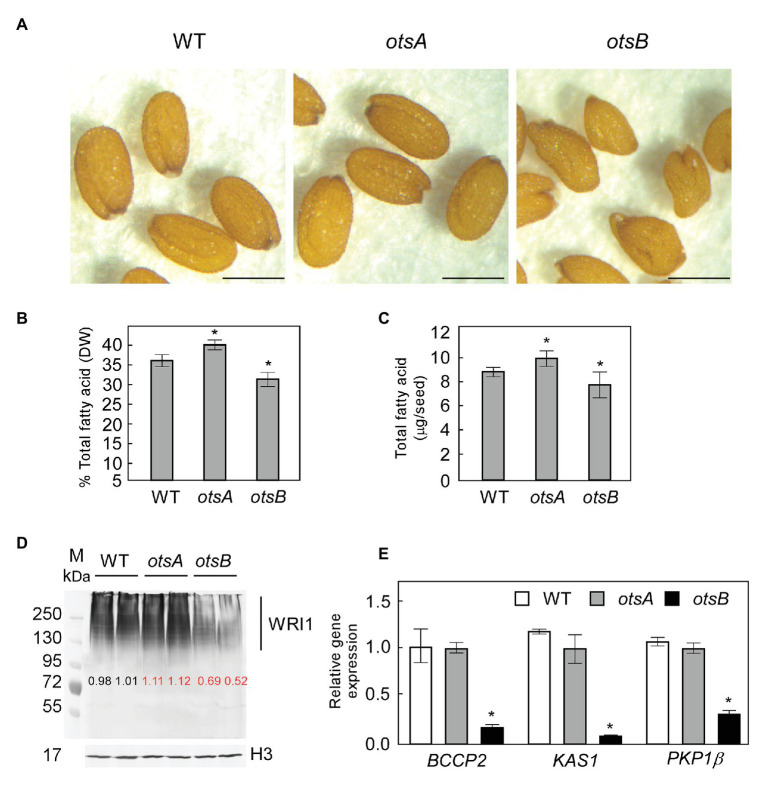
Expression of a bacterial trehalose 6-phosphate (T6P) synthase (TPS) otsA in *Arabidopsis* increases seed fatty acid content by stabilizing WRINKLED1 (WRI1). **(A)** Representative phenotype of seeds of wild type (WT) *Arabidopsis* and of *35S:otsA*- and *35S:otsB*-expressing transgenic lines. Bar = 0.2 mm. Seed total fatty acid contents are presented on percentage dry weight (DW; **B**) and per seed **(C)** basis. Values represent mean ± SD, *n* = 5 for each sample of 30 seeds. Asterisks denote statistically significant difference from WT (Student’s *t*-test, ^*^p < 0.05). **(D)** Levels of WRI1 in developing siliques [10 days after flowering (DAF)] were quantified from four biological replicates with GelAnalyzer2010 and normalized to the level of histone H3 (H3) in respective samples. A representative immunoblot is shown. Numbers in red indicate significant differences in mean levels from those of WT (Student’s *t*-test, *p* < 0.05). **(E)** Expression levels of three of the gene targets of WRI1: *BCCP2*, *KAS1*, and *PKP-1*b. Values are means ± SD (*n* = 3) from three independent experiments. Transcript abundance is expressed relative to WT. For each experiment, total RNA was isolated from siliques (10 DAF) from WT, *35S:otsA*, and *35S:otsB* transgenic plants. Asterisks denote statistically significant differences from WT [using mean crossing point deviation analysis computed by the relative expression (REST) software algorithm, ^*^p < 0.05].

In previous work, we showed that exogenous T6P can be taken up by *B. napus* suspension cells and cause a significant increase in total FA content relative to sucrose- or sorbitol-treated (Zhai et al., 2018). These experiments implicated T6P in the regulation of lipid accumulation, but its role in lipid synthesis *in planta* remained to be explored. Thus, to test whether elevated levels of T6P resulting from the expression of otsA also positively regulates FA accumulation in seeds, we obtained previously characterized transgenic *Arabidopsis* lines that constitutively express otsA with elevated T6P content, and otsB with reduced T6P content relative to WT (Schluepmann et al., 2003; Wingler et al., 2012). While 35S:*otsA* transgenic seeds appear visibly similar to those of wild type, 35S:*otsB* transgenic seeds frequently appear less symmetrical (Figure 1A). Quantification of FA showed significant differences between wild type seeds and those overexpressing either *otsA* or *otsB* (Figures 1B,C). The 35S:*otsA* transgenic seeds have 13% more FA on a DW basis than wild type seeds. Conversely, 35:*otsB* transgenic seed accumulated 12% lower levels of total FA than those of wild type (Figure 1B). Quantification of total FA as a proportion of seed dry weight showed a similar trend (Figure 1C). To detect the WRI1 polypeptide in seeds, protein was extracted from siliques 10 days after flowering (DAF) and subjected to western blotting using anti-WRI1 antibodies. Consistent with our previous analysis of *Arabidopsis* seed extracts (Zhai et al., 2017a), we detected no unmodified WRI1 (49.3 kDa), but rather an ensemble of modified WRI1 species of higher molecular masses, which were previously identified as ubiquitin-WRI1 conjugates (Figure 1D). The levels of WRI1 polypeptide in extracts of 35:*otsA* transgenic seed were approximately 10% higher than these of corresponding wild type extracts. Conversely, WRI1 polypeptide levels in 35:*otsB* transgenic seed were reduced by 40% relative to wild type. Expression levels of *BCCP2*, *KAS1*, and *PKP-β1*, three target genes of WRI1 were measured by quantitative PCR of mRNA derived from siliques 10 DAF. While significant changes were not observed in *otsA*-overexpressing transgenics at 10 DAF, all three target genes showed significantly reduced levels of expression in 35S:*otsB* transgenics compared to wild type and 35S:*otsA* transgenics at this timepoint (Figure 1E).

### Transient Expression of otsA Increases *de novo* Fatty Acid Biosynthesis in *Nicotiana benthamiana* Leaves

**Figure 2:**
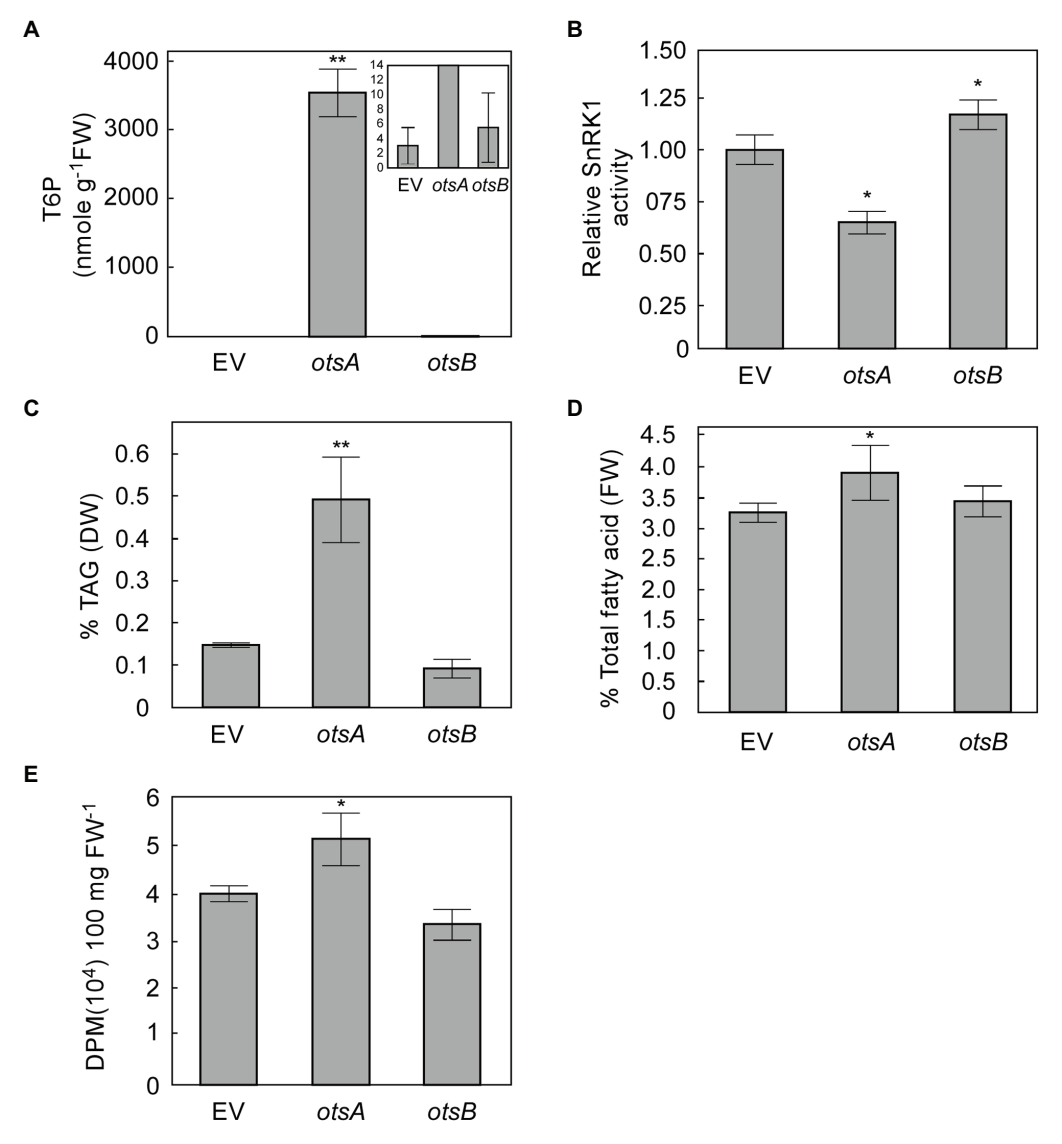
Transient expression of TPS (otsA) increases biosynthesis of both triacylglycerol (TAG) and total fatty acid in *Nicotiana benthamiana* leaves. **(A)** T6P contents in 5-week-old *N. benthamiana* leaves transformed with empty vector (EV) or expressing *otsA* or *otsB* for 3 days. Values represent mean ± SD, *n* = 5 biological replicates (Student’s *t*-test, ^*^p < 0.05; ^**^p < 0.01). **(B)** SnRK1 activity in crude extracts of infiltrated *N. benthamiana* leaves described in **(A)**. TAG **(C)** and total fatty **(D)** were quantified in the samples described in **(A)**. **(E)** [1-^14^C] acetate incorporation into fatty acyl products by strips of *N. benthamiana* leaves transiently expressing genes as indicated. Values represent mean incorporation ± SD (*n* = 5) after 30 min of labeling. Asterisks denote statistically significant differences from the EV control material (Student’s *t*-test, ^*^p < 0.05; ^**^p < 0.01).

Transient expression in *N. benthamiana* leaves has emerged as a standard model system for exploring the effects of expressing lipogenic factors in plant vegetative tissues (Wood et al., 2009; Grimberg et al., 2015). We therefore used this system to assess whether elevated levels of T6P could positively regulate FA synthesis in plant vegetative tissues. The T6P level in *N. benthamiana* leaves expressing otsA (*35S:otsA*) for 3 days significantly increased intracellular T6P by several orders of magnitude relative to empty vector (EV) controls, while T6P levels in otsB overexpressing leaves were not significantly different from those of EV controls at 3 days (Figure 2A). Extractable SnRK1 activity in leaves expressing otsA was significantly lower than EV controls, SnRK1 activity in leaves expressing otsB was significantly higher than that EV controls (Figure 2B). The otsA expressing leaves also accumulated significant 2.3-fold increases in TAG and 20% higher total FA than EV controls (Figures 2C,D). To test whether higher TAG accumulation in otsA expressing leaves resulted from *de novo* fatty acid biosynthesis, we performed [1-^14^C] acetate labeling studies in *N. benthamiana* leaves. The rate of FA synthesis in leaves expressing otsA was 29% higher than that of control leaves transformed with EV (Figure 2E).

## Discussion

The high cost of metabolic energy for lipid synthesis places an imperative on cells to proceed only when their intracellular carbon/energy levels are sufficient to support it. SnRK1, which evolved from an ancient family of energy sensors (Broeckx et al., 2016) that include mammalian AMP-dependent kinase and the fungal sucrose non-fermenting 1 (SNF1) kinase evolved to sense carbon/energy and post translationally regulate WRI1 and a host of other protein targets by phosphorylation under low carbon conditions (Zhai et al., 2017a). Such phosphorylation of WRI1 predisposes it to ubiquitination by a yet-to-be identified ubiquitin ligase leading to proteasomal degradation (Zhai et al., 2017a). Under conditions of high sugar, the levels of T6P become elevated (Lunn et al., 2006). T6P binds to KIN10, interfering with its activation by GRIK, thereby disrupting activation of SnRK1 activity limiting WRI1 phosphorylation and degradation (Zhai et al., 2018). Consequently, stabilized WRI1 accumulates and activates expression of its target genes leading to increased FA synthesis.

Optimizing TAG accumulation in plants is a central agronomic and biotechnological goal. The mechanism of WRI1 regulation detailed above offers several opportunities for intervention for optimizing TAG accumulation. One approach would be to minimize the activities of SnRK1 or its activating kinase GRIK. However, lines with strongly reduced expression of SnRK1 and GRIK activity display dwarf phenotypes that would result in unacceptable yield losses (Baena-González et al., 2007; Glab et al., 2017; Baena-González and Lunn, 2020). Another potential approach would be to interfere in other ways with the activation of KIN10 by GRIK1. In plants, a complex regulatory network maintains a proportionate relationship between the levels of T6P and the major photosynthetic sugar, sucrose (Yadav et al., 2014). While the overexpression of otsA or otsB in *Arabidopsis* does not break the correlation between sucrose and T6P, its effects can be attributed to a change in the slope of their relationship (Yadav et al., 2014). Our previous work linking T6P to the regulation of lipid synthesis was based on a combination of exogenous feeding of T6P to cultured *B. napus* cells along with biochemical and biophysical analysis. Here, we test our hypothesis that otsA-overexpressing lines containing elevated T6P (Schluepmann et al., 2003; Wingler et al., 2012) should also contain elevated levels of WRI1, FA synthesis and lipid accumulation. Conversely, the *E. coli* otsB-overexpressing lines should show reduced accumulation of WRI1, FA synthesis and lipid accumulation. That otsA overexpressing lines contained 13% more lipid and otsB overexpressing lines had 12% less lipid. Quantification of WRI1 showed that otsA-overexpressing lines had higher levels of WRI1, while otsB-overexpressing lines had lower levels of WRI1 than WT. We noted that in Figure 1E, higher levels of WRI1 did not result in higher levels of WRI1 target genes (BCCP, *KAS1*, and *PKP*). One possible explanation is that endogenous WRI1 expression peaks in seeds 10 DAF, which saturates the target gene promoters. Under such a scenario extra WRI1 resulting from its stabilization by otsA-mediated T6Pincrease would not be expected to result in an additional increase in WRI1 targets gene transcription. Taken together, these results are consistent with our hypothesis and validate the approach of using otsA overexpression to boost seed oil accumulation. However, in addition to the inhibition of SnRK1 activation by T6P, we note that constitutive otsA overexpression has been reported to result in pleiotropic effects involving changes in sucrose metabolism and its transport that could also contribute to the observed lipid phenotype (Yadav et al., 2014; Fichtner et al., 2020).

Several other approaches involving WRI1 have been explored to increase TAG accumulation. Mutations Thr70Ala and Ser166Ala, in the SnRK1 target sites within the conserved AP2 DNA-binding domains of WRI1 led to increased accumulation of theWRI1 polypeptide. However, this did not boost TAG accumulation, likely due to changes in the ability of the mutated WRI1 to bind to DNA (Zhai et al., 2017a). WRI1 was stabilized upon the expression of a 14–3-3 protein, which resulted in increased TAG accumulation, presumably through its binding to phosphorylated WRI1, blocking its recognition by the ubiquitin-conjugation complex (Ma et al., 2016). In another approach, putative N-terminal ubiquitin conjugation sites at Lys2 and Lys3 in the *Arabidopsis* WRI1 amino acid sequence were converted to Ala, resulting in both stabilization of the WRI1 polypeptide and increased TAG accumulation (Zhai et al., 2017a).

While optimizing oil accumulation in sink tissues such as seeds is desirable to maximize TAG yield per acre for conventional oilseed crops, optimizing TAG yield in vegetative source tissues of many fast-growing biomass plants, specifically in leaves (Vanhercke et al., 2017) and stems (Zale et al., 2016; Parajuli et al., 2020) has garnered much interest in recent years. Consistent with seed FA, we also observed higher total fatty acid content in leaves of *Arabidopsis* otsA-overexpressing lines than that in WT leaves (Supplementary Figure S1). However, the very low TAG contents in *Arabidopsis* leaves, makes quantifying the variation of TAG levels challenging. Because tobacco (*Nicotiana tabacum*) has been shown to tolerate leaf TAG accumulation, and *N. benthamiana* has been extensively used as a model system to study vegetative oil accumulation using transient gene expression (Wood et al., 2009; Grimberg et al., 2015), we used it to explore the effects of T6P manipulation on oil accumulation in vegetative tissues. OtsA-overexpression resulted in T6P accumulation levels several orders of magnitude higher than the reported *K*d of T6P binding to KIN10, ensuring the saturation of KIN10 by T6P, and thereby minimizing its activation by GRIK (Zhai et al., 2018). Under these conditions TAG accumulation increased by 2.3-fold, validating the otsA-overexpression approach for boosting vegetative TAG accumulation.

In summary, we show the overexpression of a bacterial T6P-synthesizing enzyme otsA has similar effects with respect to increasing TAG accumulation as we previously reported for the exogenous feeding of T6P to cultured *B. napus* cells (Zhai et al., 2018). Further, we show that otsA overexpression results in increased TAG accumulation in both sink, i.e., seed tissues, and source, i.e., leaf tissues. Deployment of otsA expression in a tissue and/or developmentally specific manner, either alone or along with other lipogenic factors (Xu and Shanklin, 2016; Vanhercke et al., 2019) may contribute to optimizing TAG accumulation in food and biofuel crops of the future.

## Data Availability Statement

The raw data supporting the conclusions of this article will be made available by the authors, without undue reservation.

## Author Contributions

JS, ZZ, and JK conceived the study. ZZ, JK, HL, RF, and JL performed experiments. ZZ, JK, RF, JL, and JS analyzed data. ZZ and JS wrote the manuscript. All authors contributed to the article and approved the submitted version.

### Conflict of Interest

The authors declare that the research was conducted in the absence of any commercial or financial relationships that could be construed as a potential conflict of interest.

## Footnotes

^1^www.thermofisher.com

^2^www.merckmillipore.com

^3^www.emdmillipore.com

^4^www.agilent.com

^5^www.arcincusa.com

^6^www.agrisera.com

^7^www.qiagen.com

## Supplementary Material

Supplementary Table S1 Oligonucleotide primer sequence pairs.
