# Supplementary figures and images for: Expression of a Bacterial Trehalose-6-phosphate Synthase otsA Increases Oil Accumulation in Plant Seeds and Vegetative Tissues
Source: Front Plant Sci. 2021 Mar 10;12:656962. doi: 10.3389/fpls.2021.656962 (PMC7988188; doi:10.3389/fpls.2021.656962)

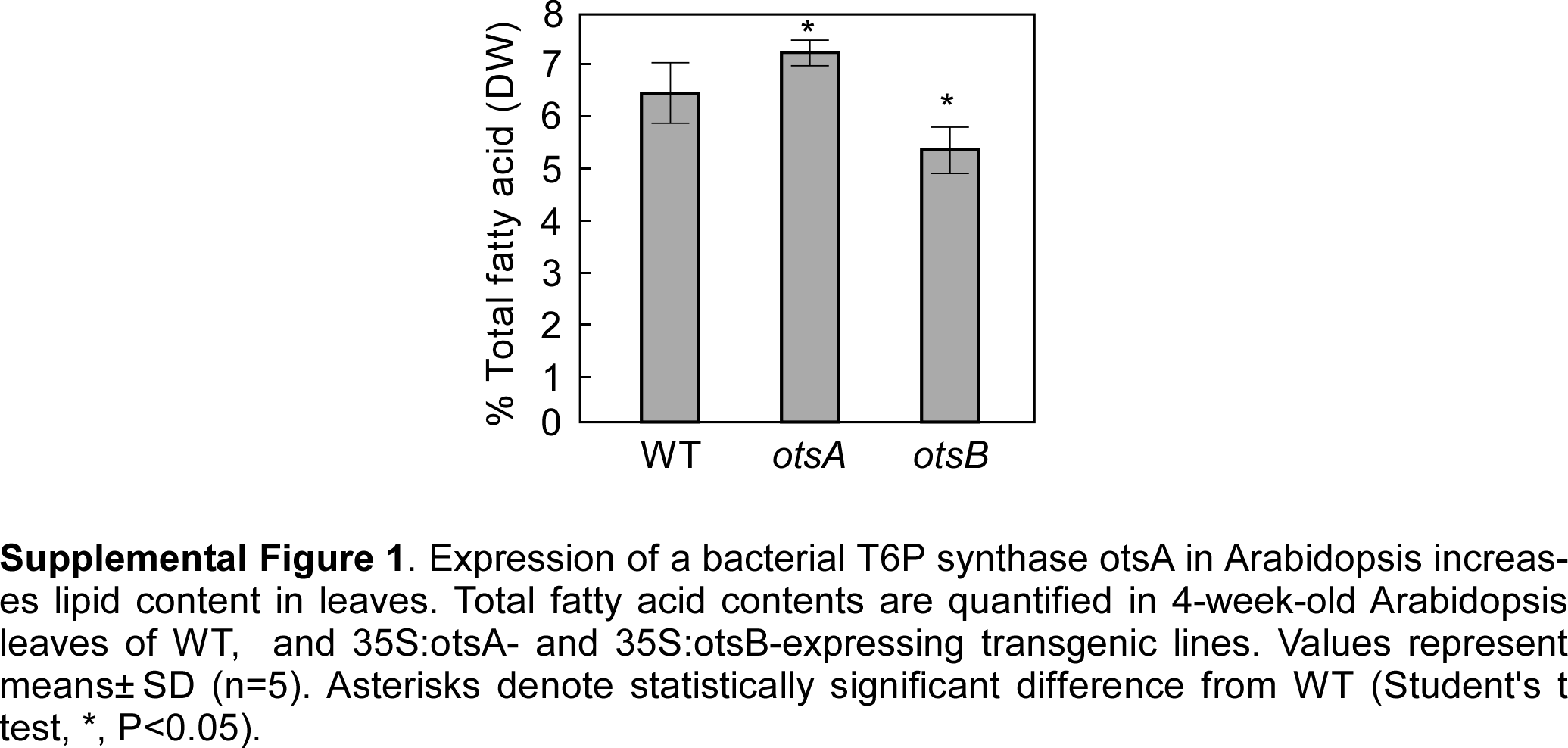

Supplement: Supplementary Table S1 — Oligonucleotide primer sequence pairs. [file Image_1.tif]
