# Supplementary material for: Expression of a Bacterial Trehalose-6-phosphate Synthase otsA Increases Oil Accumulation in Plant Seeds and Vegetative Tissues
Source: Front Plant Sci. 2021 Mar 10;12:656962. doi: 10.3389/fpls.2021.656962 (PMC7988188; doi:10.3389/fpls.2021.656962)
Supplement: Supplementary file 2 [file Table_1.pdf]

Appendix

Table S1. Oligonucleotide primer sequence pairs.

Gene	Primer Pair Sequences (forward and reverse primer, 5'-3')
otsA	GGGGACAAGTTTGTACAAAAAAGCAGGCTTCATGAGTCGTTTAGTCGTAGTATCTAACC<br>GGGGACCACTTTGTACAAGAAAGCTGGGTCTACGCAAGCTTTGGAAAGGTAGC
otsB	GGGGACAAGTTTGTACAAAAAAGCAGGCTTCATGGTGACAGAACCGTTAACCGAAACC<br>GGGGACCACTTTGTACAAGAAAGCTGGGTCTTAGATACTACGACTAAACGACTCATAGTC
BCCP2(QPCR)	AACAGCAAAACCAACATCCG<br>CCCTTCTGTACCTTATCTCCAAC
KAS1(QPCR)	CACAATTAACAGCACCTCCAAG<br>TGGGATAAAGCAAGAGATGGG
PKPβ1(QPCR)	CTCCATACCTAACTTGCACTCC<br>CTCTGCACCAAGATCACCTC
F-box(QPCR)	GGCTGAGAGGTTCGAGTGTT<br>GGCTGTTGCATGACTGAAGA
